# Olfactory Sensations During Proton and Photon Radiotherapy: A Multicenter Prospective Observational Study

**DOI:** 10.7759/cureus.22964

**Published:** 2022-03-08

**Authors:** Masashi Mizumoto, Yoshiko Oshiro, Taisuke Sumiya, Toshio Miyamoto, Keiichiro Baba, Motohiro Murakami, Shosei Shimizu, Takashi Iizumi, Takashi Saito, Hirokazu Makishima, Haruko Numajiri, Kei Nakai, Toshiyuki Okumura, Kazushi Maruo, Takeji Sakae, Hideyuki Sakurai

**Affiliations:** 1 Radiation Oncology, University of Tsukuba Hospital, Tsukuba, JPN; 2 Radiation Oncology, Tsukuba Medical Center Hospital, Tsukuba, JPN; 3 Radiation Oncology, Proton Medical Research Center, University of Tsukuba Hospital, Tsukuba, JPN; 4 Biostatistics, Faculty of Medicine, University of Tsukuba, Tsukuba, JPN

**Keywords:** smell, prospective, observational study, proton beam therapy, odor, radiotherapy

## Abstract

Purpose: Patients often report a sense of smell during radiation therapy (RT), but the details of these events are not well understood. The purpose of the study was to evaluate events of smell during photon RT and proton beam therapy (PBT).

Methods and materials: The subjects were all adult patients (≥20 years old) treated with photon RT or PBT at two centers from January 2019 to August 2020, with the exclusion of those with communication difficulties or olfactory abnormality. The presence of smell, odor type, intensity (five levels), and time period was examined prospectively using a weekly checklist.

Results: A total of 649 courses were examined in 620 patients who received photon RT (n=415) or PBT (n=205). A smell during the procedure was sensed by 51 patients (8.2%). In multivariate logistic regression analysis, nasal cavity dose (p=0.002), age (p<0.001), and photon RT (p=0.018) were identified as significant factors associated with a sense of smell. Smell occurred in only 23/515 patients (4.5%) in whom the nasal cavity was not irradiated, but in 4/19 (21.1%) and 24/86 (27.9%) with nasal cavity maximum isodose lines of 10%-50% and 60%-100%, respectively. Patients who received photon RT sensed a smell (43/415; 10.4%) more frequently than those treated with PBT (8/205; 3.9%). Of the 51 patients who sensed a smell, 32 (63%) reported a burnt smell, eight (16%) a chemical smell, two (4%) a sour smell, and nine another smell (copier machine, sweet, garbage, etc.).

Conclusions: The sense of a smell appears to be common during RT and this sensation is significantly associated with the nasal cavity dose, younger age, and photon RT.

## Introduction

Some patients who receive radiation therapy (RT) experience a smell during treatment [1]. It was assumed that this is due to the stimulation of olfactory receptors caused by ozone and free radicals that form radiochemically in the mucus on the olfactory mucosa. Recently, Hara et al. found that 68% of patients with brain or head and neck tumors experienced a visual sensation and 34% complained of olfactory sensations during RT [2]. Obinata et al. also found seven of 191 patients were aware of olfactory sensations during RT [3]. Our prospective observational study indicated that >50% of patients in whom the retina was irradiated have a visual sensation during photon RT or proton beam therapy (PBT), whereas <5% of those without retinal irradiation had a visual sensation [4]. The dose to the retina and younger patients were significant factors that showed an association with a visual sensation. However, there is very little information about odors during irradiation. Here, we examined the details of olfactory sensations during photon RT and PBT in a multicenter study with a prospective observational design. Three-dimensional conformal RT (3D-CRT), Intensity-modulated RT (IMRT), and PBT were used as RT. 3D-CRT and IMRT are classified as photon RT and irradiation is performed with the same treatment machine, and PBT is classified as particle beam therapy.

## Materials and methods

Patients and methods

**Figure 1:**
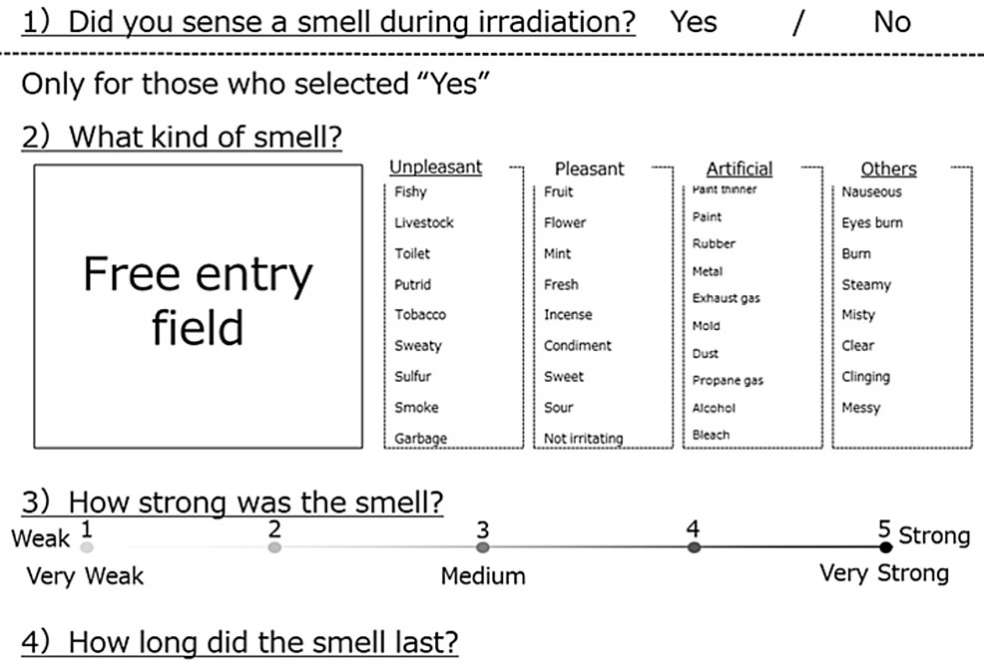
English translation of the odor sensation checklist (originally written in Japanese)

All patients aged ≥20 years old who received photon RT or PBT at two centers from January 2019 to August 2020 were enrolled in the study, except for those with communication difficulties or abnormal olfactory sensations. The study was approved by the IRB at each center (R01-160, Tsukuba Clinical Research & Development Organization; 2019-038, Tsukuba Medical Center Hospital). Both centers performed photon RT, while only one performed PBT [5-7]. The presence of smell during irradiation, type of smell, smell intensity (five levels), and time over which the smell was sensed were evaluated based on the weekly completion of a checklist. Figure 1 shows an English translation of the original checklist written in Japanese.

The evaluation items for smell were as follows: 1. Sense of smell (Yes/No); 2. What kind of smell? (Unpleasant: Fishy, Livestock, Toilet, Putrid, Tobacco, Sweaty, Sulfur, Smoke, Garbage/Pleasant: Fruit, Flower, Mint, Fresh, Incense, Condiment, Sweet, Sour, Not irritating/Artificial: Paint thinner, Paint, Rubber, Metal, Exhaust gas, Mold, Dust, Propane gas, Alcohol, Bleach/Others: Nauseous, Eyes burn, Burn, Steamy, Misty, Clear, Clinging, Messy / Free entry); 3. How strong was the smell? (1-5: 1 very weak, 3 fair, 5 very strong); 4. How long did the smell last? The dose rates of photon RT and PBT were 400-600 and 1,300 MU/min, respectively. Scattered and pulsed beams were used in PBT.

Statistical analysis

Binary (presence or absence of a smell), ordinal scale (smell intensity), and continuous (time period of smell) outcomes were evaluated using multiple logistic regression, multiple proportional odds, and multiple linear regression models, respectively, using gender, age, irradiation site (brain, head, and neck, other), nasal cavity dose, and RT type (PBT, other) as explanatory variables. P < 0.05 was considered to be significant. All statistical analyses were conducted with SAS ver. 9.4 (SAS Institute Inc., Cary, NC).

## Results

Multiple courses of irradiation were used in some patients in the study period, resulting in the examination of 649 courses for 620 patients (Table 1). Irradiation of multiple sites on the same day was defined as one course.

**Table 1:** Patient's characteristics 3D-CRT - Three-dimensional conformal radiation therapy, IMRT - Intensity-modulated radiation therapy

Characteristics	Photon (N=415)	Proton (N=205)
Age (years)	29-93 (range) 69 (median)	20-92 (range) 69 (median)
Gender		
Male	240 (58%)	165 (80%)
Female	175 (42%)	40 (20%)
Irradiated Site		
Brain	93 (22%)	12 (6%)
Head & Neck	27 (7%)	29 (14%)
Chest	146 (35%)	28 (14%)
Abdomen	18 (4%)	41 (20%)
Pelvis	125 (30%)	94 (46%)
Limbs	6 (1%)	1 (0%)
Radiotherapy Technique		
3D-CRT	296 (71%)	0 (0%)
IMRT	119 (29%)	0 (0%)
Proton	0 (0%)	205 (100%)

Photon RT (3D-CRT for 296 cases, IMRT for 119) and PBT were used in 415 patients (240 males, 175 females; median age [range] 69 (29-93) years) and 205 patients (165 males, 40 females; 69 (20-92)years), respectively. The irradiation sites included the brain (photon RT n=93, PBT n=12), head and neck (n=27, n=29), chest (n=146, n=28), abdomen (n=18, n=41), pelvis (n=125, n=94), and limbs (n=6, n=1). Overall, 51 patients (8.2%) sensed a smell during photon RT or PBT (Table 2).

**Table 2:** Rate of olfactory sensations 3D-CRT - Three-dimensional conformal radiation therapy, IMRT - Intensity-modulated radiation therapy

Characteristics	Patients	Olfactory sensations	Non-olfactory sensations	Rate (%)
Nasal Cavity				
Irradiated	105	28	77	27%
Not irradiated	515	23	492	5%
Epithelium				
Irradiated	96	26	70	27%
Not irradiated	524	25	499	5%
Age				
<50 y	69	12	57	17%
>50 y	551	39	512	7%
Gender				
Male	405	29	376	8%
Female	215	22	193	10%
Irradiated Site				
Brain	105	22	83	21%
Head & Neck	56	7	49	13%
Chest	174	14	160	8%
Abdomen	59	0	59	0%
Pelvis	219	8	211	4%
Limbs	7	0	7	0%
Radiotherapy Technique				
3D-CRT	296	36	260	12%
IMRT	119	7	112	6%
Proton	205	8	197	4%

Smells during photon RT or PBT were sensed at higher rates by patients with nasal cavity irradiation (28/105, 27%) compared to those without this irradiation (23/515, 5%); and by patients with the epithelium irradiated (26/96, 27%) compared to those who did not receive epithelium irradiation (25/524, 5%). The nasal cavity was defined as a range that included the paranasal sinus and nasopharynx, but not included the oral cavity or mesopharynx. And epithelium was defined mucosal surface of the posterior upper part of the nasal cavity.

Photon RT was administered in 43/51 patients who reported a smell (84%). These patients had a median age of 69 years and the smell was sensed by 12/69 (17%) patients aged ≤50 and 39/551 (7%) of those aged >50. With regard to tumors, smells were sensed by 22/105 (21%), 7/56 (13%), 14/174 (8%), and <5% of patients with brain, head and neck, chest, and other tumors, respectively. All of the 22 patients who reported a smell during body trunk irradiation received photon RT. Of all 51 patients, 32 (63%) reported a burnt smell, eight (16%) a chemical smell, two (4%) a sour smell, and nine another smell (copier machine, sweet, garbage, etc.). In multiple logistic regression analysis, dose to the nasal cavity (OR=1.021, p=0.002), age (OR=0.958, p<0.001), and photon RT (OR=0.358, p=0.018) were significantly associated with a sense of smell (Table 3).

**Table 3:** Multivariate logistic regression analysis of the presence of a sense of smell

Variable	Odds ratio	95% CI	P-value
Gender (female/male)	1.023	0.530-1.972	0.947
Age	0.958	0.935-0.981	<0.001
Irradiated site (brain, head & neck / other)	0.888	0.264-2.986	0.848
Nasal cavity dose	1.021	1.008-1.034	0.002
RT methods (proton/other)	0.358	0.153-0.837	0.018

Smell occurred in only 23/515 patients (4.5%) in whom the nasal cavity was not irradiated, but in 4/19 (21.1%) and 24/86 (27.9%) with nasal cavity maximum isodose lines of 10%-50% and 60%-100%, respectively. Patients treated with photon RT reported a smell (43/415; 10.4%) more frequently than those treated with PBT (8/205; 3.9%).

The median strength of the smell was 2 for all patients, and strength was significantly associated with younger age (Table 4).

**Table 4:** Multivariate logistic regression analysis of the strength of the smell

Variable	Odds ratio	95% CI	P-value
Gender (female/male)	1.079	0.319-3.650	0.903
Age	0.949	0.906-0.995	0.029
Irradiated site (brain, head, & neck/other)	3.269	0.353-30.316	0.297
Nasal cavity dose	0.991	0.970-1.013	0.419
RT methods (proton/other)	0.897	0.205-3.919	0.885

The median time of sensing a smell was 3 s (range: 1-600 s) and was <5 s in 70% of the patients (37/51). Only four patients noticed an odor sensation for ≥60 s. All four of these patients sensed a burnt smell. No factor was significantly associated with the time of the smell sensation.

## Discussion

Olfactory sensations are a well-known phenomenon in RT, with several retrospective studies showing rates of these sensations of 4% to 60% [1-3]. Sagar et al. found that 60% of patients with irradiation of the frontal lobe, whole brain, nasopharynx, pituitary fossa, and maxillary antrum experienced odorous symptoms during irradiation [1] and proposed that odor was caused by ozone since irradiation of atmospheric air produces ozone and oxides of nitrogen [8,9]. However, ozone is unstable and would only be detectable adjacent to the olfactory receptor region. Our data also indicate that including olfactory receptors (nasal cavity, epithelium) in the irradiated field is significantly associated with odor sensations: 27% of patients in whom the olfactory receptors were irradiated had a sensation of smell, whereas this occurred in only 5% in whom the olfactory receptors were not irradiated.

Ozone is known to have a burnt and disinfectant smell. In our study, 63% of patients sensed a burnt smell and 16% sensed a chemical smell; therefore, these results also suggest that odor sensations during radiotherapy are caused by ozone. Ozone is an unstable molecule that easily changes into oxygen. This may explain why the odor disappeared in a short time in most patients.

Obinata and Hara recently found that an unusual olfactory perception during RT occurred in 34% of patients who received RT at the level of the epithelium or ethmoid sinus [2,3]. Similar results were found in the current study, in which 27% of patients who received photon RT or PBT including the nasal cavity or epithelium had an odor sensation. Thus, an odor sensation seems to be felt in about 30% of cases with irradiation including the nasal cavity.

In our comparison of photon RT and PBT, patients who received photon RT had a significantly higher probability of an odor sensation. Cleland and Galloway showed that the amount of ozone generated is determined by the amount of energy absorbed by the air [10]. Our results showed that a sense of smell was infrequent among patients receiving PBT compared to photon RT. This may be because the amount of ozone produced in PBT is theoretically smaller compared to that in photon RT due to the energy peak in PBT; the so-called Bragg peak. Ionizing energy throughout the pathway in PBT is less than that in photon RT.

In our previous study on Cherenkov light during RT, younger age was significantly associated with a sense of light during RT or PBT [4]. The reason was unclear, but it may simply be that younger patients have sharper sensations that cause them to sense light and odor more frequently during RT or PBT. Many studies have found decreases in visual and olfactory sensations with age, supporting the results of this study [11-14]. Olfactory and cognitive abilities decrease with age and affect odor discrimination and identification, resulting in decreased olfactory sensation [11,12]. Color discrimination also decreases with age [13,14]. And elderly patients who need to irradiate their brains may also have some cognitive abnormalities at that time.

In this study, we found that the radiation dose to the nasal cavity had a causal relationship with odor during irradiation, but we did not assess regions other than the nasal cavity. Similar to the study of Sagar et al., it would be worthwhile to assess the relationship between odor and irradiation of regions other than the nasal cavity [1]. In addition, this study assessed subjective symptoms alone and used little objective information. In most studies, olfaction is assessed using odor samples; therefore, it is necessary to add objective assessment using questionnaires with odor samples in future studies [15].

## Conclusions

In conclusion, our results show that olfactory sensations occur during RT, and that including a nasal cavity in the irradiation field, younger age, and photon RT are significantly related to these odor sensations. We are planning a new clinical study to assess the relationship between the irradiated region and odor, with an objective assessment conducted using odor samples. This new research is expected to clarify the types of odors and the areas responsible for odors in detail.

## References

[1] Sagar SM, Thomas RJ, Loverock LT (1991). Olfactory sensations produced by high-energy photon irradiation of the olfactory receptor mucosa in humans. Int J Radiat Oncol Biol Phys.

[2] Hara N, Isobe A, Yamada K (2021). Unusual visual and olfactory perceptions during radiotherapy sessions: an investigation of the organs responsible. J Radiat Res.

[3] Obinata M, Yamada K, Sasai K (2019). Unusual olfactory perception during radiation sessions for primary brain tumors: a retrospective study. J Radiat Res.

[4] Mizumoto M, Oshiro Y, Miyamoto T (2021). Light flashes during proton and photon radiotherapy: a multicenter prospective observational study. Tech Innov Patient Support Radiat Oncol.

[5] Mizumoto M, Yamamoto T, Ishikawa E (2016). Proton beam therapy with concurrent chemotherapy for glioblastoma multiforme: comparison of nimustine hydrochloride and temozolomide. J Neurooncol.

[6] Oshiro Y, Mizumoto M, Okumura T (2012). Results of proton beam therapy without concurrent chemotherapy for patients with unresectable stage III non-small cell lung cancer. J Thorac Oncol.

[7] Mizumoto M, Okumura T, Hashimoto T (2011). Proton beam therapy for hepatocellular carcinoma: a comparison of three treatment protocols. Int J Radiat Oncol Biol Phys.

[8] Clark GL (1960). Roentgen rays: chemical effects. Med Phys.

[9] (1983). Review article. The radiological effects of nuclear war. Report of a British Institute of Radiology Working Party. Br J Radiol.

[10] Cleland MR, Galloway RA (2015). Ozone generation in air during electron beam processing. Physics Procedia.

[11] Schiffman S, Pasternak M (1979). Decreased discrimination of food odors in the elderly. J Gerontol.

[12] Wysocki CJ, Pelchat ML (1993). The effects of aging on the human sense of smell and its relationship to food choice. Crit Rev Food Sci Nutr.

[13] Uchikawa K, Sato M, Kuwamura K (2014). Effects of visual attention on chromatic and achromatic detection sensitivities. J Opt Soc Am A Opt Image Sci Vis.

[14] Paramei GV, Oakley B (2014). Variation of color discrimination across the life span. J Opt Soc Am A Opt Image Sci Vis.

[15] Miwa T, Ikeda K, Ishibashi T (2019). Clinical practice guidelines for the management of olfactory dysfunction - Secondary publication. Auris Nasus Larynx.

